# Maternal metabolizable energy intake during late gestation affects energy metabolism of the skeletal muscle of beef offspring

**DOI:** 10.1093/jas/skaf203

**Published:** 2025-06-14

**Authors:** Luiza V Kladt, Mingjia Jiang, Ziting Li, Cristina M Veloso, Koryn S Hare, Walmir Silva, Katharine M Wood, Nicola V L Serão, Mateus P Gionbelli, Michael A Steele, Marcio Duarte

**Affiliations:** Department of Animal Biosciences, University of Guelph, Guelph, ON, Canada; Department of Animal Science, Federal University of Viçosa, Viçosa, MG, Brazil; Department of Animal Biosciences, University of Guelph, Guelph, ON, Canada; Department of Animal Biosciences, University of Guelph, Guelph, ON, Canada; Department of Animal Science, Federal University of Viçosa, Viçosa, MG, Brazil; Department of Animal and Poultry Science, University of Saskatchewan, Saskatoon, SK, Canada; Department of Animal Biosciences, University of Guelph, Guelph, ON, Canada; Department of Animal Biosciences, University of Guelph, Guelph, ON, Canada; Department of Animal Science, Federal University of Lavras, Lavras, Brazil; Department of Animal Science, Federal University of Lavras, Lavras, Brazil; Department of Animal Biosciences, University of Guelph, Guelph, ON, Canada; Department of Animal Biosciences, University of Guelph, Guelph, ON, Canada

**Keywords:** energy metabolism, fetal programming, maternal nutrition, metabolic flexibility, metabolizable energy, skeletal muscle

## Abstract

The objective of this study was to evaluate the effects of varying levels of maternal metabolizable energy (ME) intake during late gestation on the changes in postnatal development of skeletal muscle in calves. A total of 42 primiparous (*n* = 21) and multiparous (*n* = 21) pregnant Angus-Simmental beef cows (680.8 ± 74.4 kg) were housed indoors at the Ontario Beef Research Center at the University of Guelph. Cows were blocked by predicted calving date, balanced by initial body weight and parity, and randomly assigned to one of 3 treatment diets designed to provide 92% (LME, *n* = 16), 104% (CME, *n* = 13), or 118% (HME, *n* = 13) of predicted ME requirements, based on a 715 kg Angus cow with body condition score (BCS) 7 programmed to lose 2 BCS units over late gestation. All cows were managed under a planned moderate negative energy balance starting 53 d before expected calving. At birth, calves were weighed before suckling the dam, and again on day 209 at weaning. At 28 d of age, plasma and serum samples were collected for insulin, glucose, BHBA, and non-esterified fatty acids analysis. At 30 d of age *Longissimus* muscle samples were biopsied from the calves and were used for mRNA expression and protein abundance for energy metabolism. All statistical analyses were performed in SAS Studio, in a mixed model including the fixed effects of treatment and parity, and the random effect of sire. No differences were observed among treatments for calf birth weight, weaning weight, and metabolic profile. A lower mRNA expression of *MYH1* was observed (*P* = 0.02) in the skeletal muscle of calves from the HME and LME groups compared to the CME. An increased mRNA expression of both *MYH2a* (*P* = 0.04) and *MYH2 × *(*P* = 0.01) was observed in calves from LME compared to HME, suggesting potential alterations in muscle fiber composition that may influence metabolic efficiency and growth performance. A greater mRNA expression of *PPARα* (*P* = 0.04), *PPARGC1α* (*P* = 0.04), and *MEF2A* (*P* = 0.01) were observed in calves from the LME group compared to the HME. A greater AMPK activity was observed (*P* = 0.01) in the skeletal muscle of calves from the LME group compared to CME and HME. In contrast, Akt activity was greater in HME and LME groups compared to CME (*P* = 0.01). Our findings suggest that maternal ME intake affected the muscle energy metabolism of the offspring, the oxidation of fatty acids, mitochondrial biogenesis, and the use of muscle fiber-type fuel.

## Teaser

Cow’s metabolizable energy (ME) intake during late gestation influenced the energy metabolism of offspring muscle. Providing 92% of the ME requirement enhanced skeletal muscle fatty acid oxidation, promoted mitochondrial biogenesis, and increased the prevalence of muscle fibers with the capacity to adapt their fuel utilization.

## Introduction

Proper maternal nutrition during late gestation is crucial for fetal development and the long-term productivity of the offspring (Funston et al., 2010; Costa et al., 2021; Santos et al., 2022). While previous research indicates that increasing maternal energy density during late gestation may be useful to improve the energy status of cows (Barcelos et al., 2022; Waldon et al., 2023), a low-energy diet negatively impacts the growth and development of neonatal calves (Chen et al., 2022). Other studies have shown that energy restriction during early development can alter muscle and blood transcriptomes, potentially affecting muscle development and metabolic pathways (Sanglard et al., 2018; Costa et al., 2021; Nascimento et al., 2024).

Maternal nutrition during gestation can also influence the skeletal muscle metabolism of offspring, driving structural and functional changes in muscle that support increased metabolic flexibility postnatally (Norman et al., 2012; Carvalho et al., 2022). While muscle fiber types begin to form in the embryonic phase, their phenotype can change after birth. This ability of skeletal muscle to adjust its energy metabolism is referred to as “fiber plasticity” (Talbot and Maves, 2016), which allows the tissue to switch between fuel sources. The regulation of this process involves signaling pathways that control key aspects of metabolism, including glucose utilization, lipid oxidation, and mitochondrial biogenesis (Lin et al., 2002). This inability to rapidly adjust energy substrate utilization has been termed metabolic inflexibility and has recently been associated with metabolic chronic diseases (Shoemaker et al., 2023). Studies in rodents have shown that nutrient restriction during gestation can enhance oxidative metabolism in offspring under ad libitum conditions. However, it may also impair their ability to adapt fuel usage in skeletal muscle (Aragão et al., 2014). Additionally, offspring born to nutrient-restricted dams showed gene expression patterns in skeletal muscle that promote the transport of long-chain fatty acids into mitochondria, indicating a preference for specific carbon sources for energy production (Aragão et al., 2014). These findings indicate that maternal nutrition exerts a programming effect on offspring skeletal muscle metabolism, potentially influencing the preferential utilization of specific carbon substrates for energy production. Consistent with this, Meneses et al. (2024) demonstrated that protein supplementation during mid-gestation enhances fetal growth and optimizes nutrient utilization, thereby further substantiating the role of maternal dietary composition in the metabolic programming of offspring. However, investigations into these mechanisms in cattle remain limited and additional research is warranted to elucidate the effects of maternal energy intake on the developmental programming of skeletal muscle energy metabolism in offspring.

In this study, we hypothesize that maternal metabolizable energy (ME) intake during late gestation programs skeletal muscle metabolism in offspring by modulating the expression of genes and key proteins involved in oxidative metabolism and muscle fiber type. Specifically, we propose that energy intake restriction enhances fatty acid oxidation and the expression of oxidative markers in offspring skeletal muscle, in comparison to offspring born to dams fed at maintenance and above maintenance levels during late gestation. As such, this study aims to explore how varying levels of maternal ME intake during late gestation impact the skeletal muscle energy metabolism in beef offspring.

## Material and Methods

All procedures used in this study were approved by the animal care committee at the University of Guelph (Animal Utilization Protocol No. 4419) and followed the guidelines of the Canadian Council on Animal Care (CCAC, 1993) .

### Animals, treatments, and experimental design

Forty-two pregnant Angus-Simmental beef cows (680.8 ± 74.4 kg), including primiparous (*n* = 21) and multiparous (*n* = 21), were utilized and housed at the Ontario Beef Research Center at the University of Guelph. Cows were artificially inseminated with semen from 7 Black Angus bulls of similar genetic merit. Heifers were bred to begin calving 2 wk earlier than the cows. Multiparous cows had between 2 and 3 previous parturitions. Cows were blocked by predicted calving date based on an estimated gestation period of 282 d and treatments were balanced by initial body weight (IBW), sire, and parity.

Experimental diets (Table 1) were formulated using CNCPS 6.55 (Nutritional Dynamic System software, RUM&N Sas, Via Sant’ Ambrogio, Italy) to deliver the specified ME levels while meeting or exceeding metabolizable protein (MP) requirements ( Higgs et al., 2015; Van Amburgh et al., 2015a, b). Model inputs were based on herd averages from the Ontario Beef Research Center, encompassing both heifers and cows. This approach reflects common industry practices in Canadian cow–calf systems and addresses logistical constraints that prevented rationing by parity. Predicted ME and MP requirements were calculated using a reference model cow: a 715 kg Angus female with a body condition score (BCS) of 7, programmed to lose 2 BCS units over the final 100 d of gestation and calving a 38 kg calf. This scenario represents a planned moderate negative energy balance and served as the baseline to define 100% of ME requirements. Accordingly, cows were randomly assigned to 1 of the 3 dietary treatments designed to provide 92% (LME, *n* = 16), 104% (CME, *n* = 13), or 118% (HME, *n* = 13) of the predicted ME requirements. Therefore, CME and HME represent relative increases over a restricted baseline, not necessarily indicative of BCS maintenance or gain.

**Table 1.** Ingredient composition, chemical characteristics, and feeding rates of prepartum diets providing 118% (HME), 104% (CME), and 92% (LME) of predicted metabolizable energy (ME) requirements for pregnant cows from day −53 to calving, and ad libitum postpartum lactation diet

Item	Experimental treatments			
	HME	CME	LME	Lactation
Diet composition, %DM				
Haylage	5	5	5	32.1
Corn silage	5	5	5	25.2
Chopped dry hay	44.5	37.5	30.5	0
Wheat straw	5	30	55	37.8
High moisture corn	18.5	18.5	0	0
Whole corn	18.5	0	0	0
Urea	0	0.5	1	0
Vitamin and mineral premix^1^	3.5	3.5	3.5	5.0
DM, %	80.76	80.86	84.53	51.1
Chemical composition, %DM				
Crude protein	10.83	10.62	10.58	9.6
aNDFom	36.78	51.91	67.38	54.93
Starch	26.98	14.95	2.87	9.29
Ether extract	3.02	2.57	2.16	2.68
Ca	0.66	0.66	0.66	0.68
P	0.41	0.34	0.27	0.32
Mg	0.26	0.25	0.24	0.27
K	1.64	1.57	1.51	1.44
Predicted ME supply, Mcal/kg	2.47	2.26	2.14	1.92
Predicted ME supply, % requirement	117.5	104.1	92.4	151.3
Predicted MP supply, % requirement	124.9	120.5	113.7	166

^1^The mineral–vitamin premix was formulated by Floradal Feed Mill Limited (Floradale, ON); chemical composition: 293.4 kIUU/kg vit A, 67.1 kIU/kg vit D, 1364.6 kIU/kg vit E, 447.0 ppm Cu, 1586.4 ppm Fe, and 3866.6 ppm Zn

Experimental diets began at 53 ± 0.4 d before expected calving. Cows were housed by treatment and parity in pens of 6 animals, sharing 3 Insentec feeders (Roughage Intake Control System, Insentec, Marknesse, the Netherlands), and were limit-fed at 1.35% (LME), 1.4% (CME), or 1.45% (HME) of body weight on a dry matter basis. Feed quantities were adjusted weekly based on ingredient dry matter and cow body weight. Prior to diet implementation, cows underwent a 2-wk adaptation period receiving the CME diet to acclimate to limited feeding. Postpartum, all cows were fed a common lactation diet ad libitum.

Calving occurred during spring (early February to early May). After birth, cow–calf pairs were moved to individual maternity pens for 2 d to support bonding, then housed in groups with access to a creep pen for clean bedding. Calves suckled ad libitum until weaning. At 60 d postpartum, pairs were moved to pasture as a single herd, grazing on a mix of grasses and legumes. Calves were weaned at 209 d of age. Forages were sampled twice weekly, concentrates were sampled every 2 wk, and total mixed rations were sampled weekly. Feed samples were dried in a gravity convection oven at 60°C for 2 consecutive days (dry forages and concentrates) or until consistent weights were recorded across 2 calendar dates (ensiled forages). Dry matter analysis of feed ingredients was used to adjust rations for any changes in dry matter using a running average across the study. After the study, dried samples were ground through a 1-mm screen and were sent to A&L Canada Laboratories Inc. (A&L Canada Laboratories Inc., London, ON) for wet chemistry analysis. Samples were analyzed using the AOAC methodology (AOAC, 1990) . Neutral detergent fiber and acid detergent fiber were measured with the Ankom 200 using the Ankom Method 6 and Ankom Method 5 procedures (AOAC 2002.04, AOAC 973.18). Crude protein was measured by combustion with the LECO FP628 nitrogen analyzer (AOAC 990.03). Crude fat (EE) was measured with petroleum ether as a high-temperature solvent to extract fat and oil (AOAC 920.39). Starch was measured using heat-stable amylase and amyloglucosidase with the Megazyme K-TSTA method (AOAC 996.11). Sugar was measured using sulphuric acid/phenol and 80% ethanol:water using the colorimetric method.

### Animal data and sample collections

Cows’ IBW was collected on 2 consecutive days at treatment enrollment, on days −54 ± 0.4 and −53 ± 0.4 relative to parturition. Final body weight (FBW) was measured on day −3 ± 0.4 before parturition. Initial subcutaneous fat thickness (ISFT) was measured at both the rib and rump using ultrasonography with an EXAGO ultrasound machine and a linear transducer (#L3180B, 18 cm, 3.5 MHz frequency, Echo Control Medical, Angoulême, France) on day −54 ± 0.4 relative to parturition. Additionally, final subcutaneous fat thickness (FSFT) was measured again at the rib and rump on day 13 ± 0.3 after parturition. The Rib fat thickness was measured at the ¾ end of the ribeye area, using an image collected between the 12th and 13th ribs. The rump fat thickness was assessed at the rump, between the ischium and the pubis. Ultrasound image analysis was performed using ImageJ (National Institutes of Health, Maryland, USA). Initial and final body weights (BW) were used to calculate total dry matter intake (DMI) and as a percentage of body weight during the prepartum period. Similarly, body weight recorded after parturition and on day 55 ± 0.3 postpartum was used to calculate total DMI and intake as a percentage of body weight during the postpartum period. The variation in subcutaneous fat thickness was obtained relative to the measurements at the prepartum period as follows:


$$\begin{array}{l} VSFT=\;\frac{FSFT-ISFT}{ISFT}\;\times \;100 \end{array}$$

Where VSFT (%) is variation in subcutaneous fat thickness; FSFT is final subcutaneous fat thickness; and ISFT is initial subcutaneous fat thickness.

At birth, calves were weighed before suckling the dam, and again on day 28 ± 0.18, and day 209 ± 1.38 at weaning. The birth and weaning weights were used to calculate the average daily gain (ADG) of the calves during the suckling phase, body weight gain from birth to weaning divided by days of age at weaning. At 28 d of age, blood samples were collected from the calves by jugular venipuncture in 10 mL green top heparinized saline vacutainer tubes and red top vacutainer tubes. (BD Vacutainer®, BD and Company, Franklin Lakes, New Jersey, USA). Aprotinin (Aprotinin from bovine lung, Sigma Aldrich, St. Louis, Missouri, USA) was added to plasma samples at a ratio of 0.5 μL per 1 mL of blood. These samples were immediately placed on ice after collection. Serum samples were allowed to clot at room temperature. Plasma and serum samples were centrifuged at 2,500 × *g* (4 °C) for 15 min. After centrifugation, supernatant from the samples was separated and stored at −20 °C until analysis.

At 30 d of age Longissimus dorsis muscle samples (located between the 12th and 13th ribs) were biopsied from the calves. The biopsy site was initially shaved and sanitized with 4% chlorohexidine and 70% ethanol alcohol. Following the sanitization, the biopsy site was anesthetized with 2% lidocaine (7 mL). A 1.5-cm incision was made through the skin using a 14-blade scalpel and muscle tissue was excised by using an 8 mm biopsy punch and rinsed with 0.9% Saline solution. The skin was sealed using veterinary surgical glue (VetBond, 3M). The muscle samples were rinsed with a sterile saline solution, placed in a 2mL cryotube, and immediately snap-frozen in liquid nitrogen for transport to the lab. In the lab, the samples were powdered in liquid nitrogen using a pre-cooled mortar and pestle, transferred to a pre-cooled cryotube, and stored at −80 °C for future nucleic acid and protein extraction.

### Plasma and serum analyses

Serum samples were analyzed for the Bovine Metabolic Profile at University of Guelph’s Animal Health Laboratory (Animal Health Laboratory, University of Guelph, Guelph, ON) photometric analysis using an autochemistry analyzer (Cobas 6000 c501, Roche Diagnostics, Indianapolis, IN) including glucose, beta-hydroxybutyrate (BHBA) and non-esterified-fatty-acids (NEFA).

Plasma samples were analyzed for insulin using the ALPCO Bovine Insulin ELISA kit (ALPCO, Salem, NH, USA) following the manufacturer’s instructions: plates were read once at 450 nm using an absorbance reader (Cytation 5, BioTek, Winooski, VT, United States). Samples were analyzed in duplicate with and inter-assay CV of 4.6% and an intra-assay CV of 2.9%.

### Total RNA extraction and mRNA expression analyses

Total RNA was extracted from 0.1 g of tissue using Trizol® (InvitrogenTM, Thermo Fisher Scientific®, Oregon, USA) following the manufacturer’s recommendations. The total RNA was quantified with a NanoDrop spectrophotometer (Thermo Fisher Scientific®, Waltham, MA, USA), ensuring an optimal 260/280 ratio between 1.8 and 2.0, and the integrity was assessed in a 1% agarose gel. The RNA samples were reverse transcribed into cDNA using the GoScriptTM Reverse Transcription System Kit (Promega, Madison, WI, USA). The primers (Table 2) for amplification of target and endogenous genes were designed using PrimerQuest Software (PrimerQuest–design qPCR assays | IDT [idtdna.com]) with sequences obtained from GenBank (GenBank Overview [nih.gov]). Real-time quantitative PCR was performed in the thermal cycler QuantStudio 3 (Applied Biosystems, Foster City, CA, USA) using the SYBR Green detection method (Applied Biosystems, Foster City, CA, USA) and SYBR™ Green PCR Master Mix (InvitrogenTM, Thermo Fisher Scientific®, Oregon, USA). The amplification efficiency was 0.90 to 0.99. After amplification, a melting curve (0.01 °C/s) was used to confirm product purity and to confirm the specificity of the primers, ensuring that only the intended target sequence was amplified. The results of gene expression were calculated according to the methods described by Steibel (2009).

**Table 2.** List primers for mRNA expression by RT-qPCR

Gene symbol	NCBI accession number	Primer
*Target genes*		
*ACACA*	NM_174224.2	F: CTCCAACTTCCTTCACTCCTTAG R: ACATACTTCACTCCCTCGTAGA
*CPT1A*	NM_001304989.2	F: CCCAAATCATGCACTGTTGAC R: CCTGAGAGACAAGCCCAAATAG
*FASN*	NM_001012669.1	F: AGCACACGCCTGTAGTATTC R: TTCCAGAAGCCGCACTTT
*IRS1*	XM_003585773.6	F: GCCTATGCCAGCATCACTTT R: GGAGGATTTGCTGAGGTCATTT
*MEF2A*	NM_001083638.2	F: TCCACCTCAAGCCACATTAC R: GAGGTCTGAAGTGCTCAACAT
*MYH1*	NM_174117.1	F: GTACGTGAACTGGAAGGAGAAG R: AAATCCTGGAGCCTGAGAATG
*MYH2a*	NM_001166227.1	F: AGAGCAGAGGATGAGGAAGA R: GTCAGCTCAAGGTCGTCTATG
*MYH2x*	XM_010816053.4	F: CCTTACCTCCGAAAGTCTGAAA R: CTGCTCTGGATAGTCCCTTTG
*MYH7*	NM_174727.1	F: CAAGGGCTTGAATGAGGAGTAG R: GCTTTATTCTGCTTCTTCCAAAGG
*PPARα*	NM_001034036.1	F: CCTACGGGAATGGCTTCATAAC R: GCAGCCACAAAGAGGGAAATA
*PPARγ*	NM_181024.2	F: TTATTCCCACCTCCTCCAAAC R: CACGACTCCCACCGATATTT
*PPARGC1α*	NM_177945.3	F: ACACCAAACCCACAGAGAAC R: GGGATGACCGAAGTGCTTATT
*SLC2A4*	NM_174604.1	F: CTTGGTCCTTGGCGTATTCT R: CAGGTCTCATTGTAGCTCTGTT
*SREBF1*	NM_001113302.1	F: GACTACATCCGCTTCCTTCAG R: CCAGGTCCTTCAGCGATTT
*UCP3*	NM_174210.1	F: GGTGAAGACGCGGTATATGAA R: CAAAGCACTGAAGACCACAATC
*Endogenous gene*		
*18s*	NM_001304989.2	F: CCCAAATCATGCACTGTTGAC R: CCTGAGAGACAAGCCCAAATAG

### Protein extraction and western blotting analyses

Total protein was extracted from 0.1 g of tissue in 1 mL of lysis buffer (10 mM of Tris HCl [pH 7.6], 150 mM of NaCl, 1% of Triton X-100, 0.5% sodium deoxycholate, 1% sodium dodecyl sulfate [SDS], and 1% of protease inhibitor cocktail mammalian cells and tissues [Sigma-Aldrich®]), and the lysate was sonicated. Total protein content was estimated using the Bradford protein assay (Bio-Rad, Hercules, CA, USA), aliquoted and stored at −80 °C. The proteins were separated using a 10% SDS-PAGE gel loaded with 40 μg of protein per sample, transferred to a 0.45 μm Nitrocellulose membrane (Bio-Rad, Hercules, CA, USA) using a semi-dry Trans-Blot Turbo Transfer System (Bio-Rad, Hercules, CA, USA), and blocked for 1 h at room temperature with 3% bovine serum albumin (BSA, Sigma Aldrich®) in 1x Tris-Buffered Saline (TBS1x; 50 mM Tris-HCL, pH 7.5; 150 mM NaCl; Sigma Aldrich®). Subsequently, the membranes were incubated for 12 h at 4 °C with the primary antibodies (Table 3) diluted in a blocking solution. After 12 h of incubation, membranes were washed 3 times 5 minutes with Tris Buffered Saline and 0.1% Tween® (TBSt) and incubated with the secondary antibody (Table 3) diluted in blocking solution for 1 h at room temperature. Membranes were then washed with TBSt 3 times 7 min, revealed by ECL Plus Western Blotting Detection System (GE HealthCare, Buckinghamshire, Ul) and the images generated by ChemiDoc XRS + System (Bio-Rad, Hercules, CA, USA) and evaluated using Image Lab Software (Bio-Rad, Hercules, CA, USA). The α-tubulin was used as a housekeeping protein to normalize the abundance of the target proteins.

**Table 3.** List of antibodies used in Western blotting analysis

Antibody	Host	Dilution	Manufacturer	Catalog number
*Primaries antibodies*				
Akt	Rabbit Monoclonal IgG	1:1000	Cell signaling	9272S
p-Akt	Rabbit Monoclonal IgG	1:1000	Cell signaling	9271S
AMPK	Rabbit Monoclonal IgG	1:1000	Cell signaling	2532S
p-AMPK	Rabbit Monoclonal IgG	1:1000	Cell signaling	2535S
α-tubulin	Rabbit Monoclonal IgG	1:1000	Cell signaling	2144S
*Secondary antibodies*				
HRP	Goat anti-Rabbit IgG	1:5000	Thermo Fisher Scientific®	A16096

### Statistical analysis

The dam and calf performance traits and calf metabolism traits were analyzed according to the following mixed model:


$$\begin{array}{l} Y_{jklm}=\mu +T_{j}+P_{k}+S_{l}+e_{jklm} \end{array}$$ (1)

where $Y_{jklm}$ is the observed value; μ is the intercept; $T_{j}$ is the fixed effect of the *j*^th^ level of maternal dietary treatment, with *j* = 1…3 $P_{k}$ is the fixed effect of the *k*^th^ level of parity, with *k* = 1…2; $S_{l}$ is the random effect of the *l*^th^ Sire, with *l* = 1…7 assuming $S∼N(0,I_{S}\sigma_{S}^{2})$, where $I_{S}$ represents the identity matrix of dimension equal to the number of sires; and $e_{jklm}$ is the random error associated with $y_{jklm}$, assuming $e∼N(0,I_{e}\sigma_{e}^{2})\;$ where $I_{e}$ represents the identity matrix of dimension equal to the number of observations. Prior to final analyses, residuals were evaluated for normality. For each analysis, data points were removed one at a time until absolute Studentized residuals were lower than 3 and had nonsignificant (*P* > 0.01) Shapiro–Wilk’s test for normality. Expected means were generated from the final models and separated using Tukey’s test when different at *P *≤ 0.05.

The protein abundance data were analyzed using a Poisson mixed model using a log-link function:


$$\begin{array}{l} Y_{jklm}=e^{\mu +T_{j}+P_{k}+S_{l}} \end{array}$$ (2)

where e is the exponential, and $Y_{jklm}$, μ, $T_{j}$, $P_{k}$, and $S_{l}$ are as previously defined in Equation 1. Expected rates were generated from the final models and separated using Tukey’s test when different at *P *≤ 0.05.

Prior to the analysis of the RT-PCR data, the CT values were adjusted (adjCT) for their respective primer efficiency and back-transformed to the $log_{2}$ scale as:


$$\begin{array}{l} adjCT=\log_{2}(E^{-CT}) \end{array}$$ (3)

where E=1+Eff, where Eff represents the calculated primer efficiency. Afterward, the adjCT data were analyzed according to the linear mixed model below, following the strategy proposed by Steibel et al., 2009 where the data on the target and endogenous control genes are analyzed simultaneously:


$$\begin{array}{lll} adjCT_{ijklm}=\; & \mu +G_{i}+T_{j}+{\left(G*T\right)}_{ij}\; & +P_{k}+{\left(G*P\right)}_{ij}+S_{il}+A_{jklm}+e_{ijklm} \end{array}$$ (4)

where μ, $T_{j}$, and $P_{k}$ are as previously defined, whereas $adjCT_{ijklm}$ is the adjusted CT as in Equation 3, $G_{i}$ is the fixed effect of the *i*^th^ level of gene, with *i* = 1,2; ${\left(G*T\right)}_{ij}$ is the fixed effect for the interaction between $G_{i}$ and $T_{j}$; ${\left(G*P\right)}_{ik}$ is the fixed effect for the interaction between $G_{i}$ and $P_{k}$; $S_{il}$ is the random effect of the *l*^th^ Sire on each gene, assuming $S∼N(0,I_{GS}\sigma_{S_{i}}^{2})$, where $I_{GS}$ represents the identity matrix with dimensions equal to the number of sires times the number of genes (i.e., 2); $A_{jklm}$ is the random effect of the *m*^th^ animal, assuming $A∼N(0,I_{A}\sigma_{S_{i}}^{2})$, where $I_{A}$ represents the identity matrix with dimensions equal to the number of animals; and $e_{ijklm}$ is the random error associated with $adjCT_{ijklm}$, assuming $e∼N(0,I_{Ge}\sigma_{e}^{2})$, where $I_{Ge}$ represents the identity matrix with dimensions equal to the number of observations across the 2 genes. The interactions between $G_{i}$ with $T_{j}$ and $P_{k}$, respectively, were used to assess the effects of *Treatment* and *Parity*, respectively, through orthogonal contrasts, as in Steibel et al. (2009). Expected adjCT were computed as −ΔadjCT, such that the differences between the levels of *Treatment* and *Parity* were estimated as −ΔΔadjCT, and then used to compute the *Gene Ratio Expression* (GRE) as:


$$\begin{array}{l} GRE=2^{-\Delta \Delta CT} \end{array}$$ (5)

Estimates of *GREs* were separated using contrasts when different at *P *≤ 0.05. All analyses were performed in SAS Studio 3.81 (Enterprise Edition, SAS Institute Inc., Cary, NC, USA).

## Results

### Cow–calf performance and calf metabolic parameters

The IBW (*P* = 0.26) did not differ among treatments. However, the final body weight (FBW; *P* = 0.01) was greater in the HME and CME compared to the LME cows (Table 4). The initial subcutaneous fat thickness at the 12th and 13th ribs (ISFT-rib; *P* = 0.40) and at the rump (ISFT-rump; *P* = 0.12) did not differ between treatments (Table 4). However, the final subcutaneous fat thickness at the rib (FSFT-rib; *P* = 0.01) and at the rump (FSFT-rump; *P* = 0.04) was greater in the HME compared to LME cows. The variation in subcutaneous fat thickness at the rib (VSFT-rib; *P* = 0.01) and at the rump (VSFT-rump; *P* = 0.05) was lower in HME than in LME cows (Table 4). The ADG (*P* = 0.01) before calving was lower in LME compared to CME and HME cows (Table 4). During the prepartum period, cows fed the HME and CME diets had greater (*P* < 0.01) DMI than those fed the LME diet, as designed. Prepartum DMI per percentage of body weight percentage (%BW, *P* < 0.01) was greater in cows fed the HME and CME diets than in those fed the LME diet (Table 4). In the postpartum period, total DMI (*P* = 0.01) and DMI per %BW (*P* = 0.02) were higher in the CME treatment compared to HME (Table 4).

**Table 4.** Least-square means ± SEM for the performance of cows and calves born to dams fed prepartum diets providing 118% (HME), 104% (CME), and 92% (LME) of the predicted metabolizable energy (ME) requirements for pregnant cows from day −53 to calving, followed by an ad libitum postpartum lactation diet

Item	Experimental treatments			*P*-value
	HME	CME	LME	Treatment
*Cow performance*				
Initial body weight, kg	664.5 ± 18.9	706.9 ± 18.9	673.5 ± 16.9	0.26
^ 1^Final body weight, kg	713.8 ± 6.4 ^a^	710.8 ± 6.0 ^a^	672.4 ± 5.8 ^b^	0.01
Initial subcutaneous rib fat thickness, mm	9.29 ± 0.85	8.26 ± 0.84	7.75 ± 0.75	0.40
Final subcutaneous rib fat thickness, mm	7.30 ± 0.41 ^a^	7.31 ± 0.41 ^a^	5.86 ± 0.37 ^b^	0.01
Variation in subcutaneous rib fat thickness, %	−14 ± 0.05 ^a^	−6 ± 0.05 ^a^	−32 ± 0.04 ^b^	0.01
Initial subcutaneous rump fat thickness, mm	16.42 ± 1.51	13.29 ± 1.49	12.31 ± 1.32	0.12
Final subcutaneous rump fat thickness, mm	13.51 ± 0.48 ^a^	12.51 ± 0.49 ^ab^	11.50 ± 0.45 ^b^	0.04
Variation in subcutaneous rump fat thickness, %	−5 ± 0.01 ^a^	−11 ± 0.05 ^ab^	−19 ± 0.05 ^b^	0.05
Average daily gain prepartum, kg/day	0.63 ± 0.12 ^a^	0.57 ± 0.12 ^a^	−0.19 ± 0.11 ^b^	0.01
Dry matter intake prepartum, kg/day	9.81 ± 0.15 ^a^	9.75 ± 0.15 ^a^	8.48 ± 0.11 ^b^	0.01
Dry matter intake prepartum, %BW	1.48 ± 0.02 ^a^	1.45 ± 0.02 ^a^	1.29 ± 0.02 ^b^	0.01
Dry matter intake postpartum, kg/day	14.95 ± 0.48 ^b^	17.13 ± 0.50 ^a^	15.64 ± 0.46 ^ab^	0.01
Dry matter intake postpartum, %BW	2.15 ± 0.08 ^b^	2.40 ± 0.08 ^a^	2.29 ± 0.08 ^ab^	0.02
*Calf performance*				
Birth weight, kg	34.67 ± 1.3	35.28 ± 1.3	33.42 ± 1.2	0.52
Weaning weight, kg	302.62 ± 9.57	275.11 ± 9.18	283.86 ± 8.04	0.13
ADG to 209 d, kg/d	1.23 ± 0.06	1.18 ± 0.06	1.21 ± 0.06	0.75

^1^Adjusted for initial body weight.

^2^Final body weight (FBW) and subcutaneous fat thickness (FSFT) were recorded 3 d prior to the expected calving date.

a-b Means within a row lacking a common superscript letter differ at *P* < 0.05.

No differences were observed among treatments for calf birth weight (*P* = 0.52), weaning weight (*P* = 0.13), or ADG (*P* = 0.75) as shown in Table 4. No differences were observed between experimental treatments on serum levels of insulin *(P* = 0.47), glucose (*P* = 0.92), β-hydroxybutyrate (BHBA; *P* = 0.72), and non-esterified fatty acids (NEFA; *P* = 0.68) in calves at 28 d of age (Table 5).

**Table 5.** Least-square means ± SEM for blood metabolic parameters of calves born to dams fed prepartum diets providing 118% (HME), 104% (CME), and 92% (LME) of the predicted metabolizable energy (ME) requirements from day −53 to calving, followed by an ad libitum postpartum lactation diet. Measurements were taken when the calves were 28 d old

Item	Experimental treatments			*P*-value
	HME	CME	LME	Treatment
Insulin, μg/L	3.48 ± 0.55	4.43 ± 0.55	3.70 ± 0.49	0.47
Glucose, mmol/L	6.54 ± 0.22	6.44 ± 0.22	6.54 ± 0.20	0.92
BHBA, μmol/L	66.36 ± 7.86	61.47 ± 7.86	70.0 ± 7.03	0.72
NEFA, mmol/L	0.13 ± 0.01	0.14 ± 0.01	0.15 ± 0.01	0.68

BHBA, beta-hydroxybutyrate; NEFA, non-esterified fatty acids.

### mRNA expression of energy metabolism in the skeletal muscle of the offspring

The mRNA expression of *IRS1* (*P* = 0.74) and *SLC2A4* (*P *= 0.92) in the skeletal muscle of the offspring did not differ between treatments, while a greater mRNA expression of *MEF2A* was observed in the LME group compared to HME and CME groups (*P* < 0.01; Table 6). The mRNA expression of *MYH1* was decreased (*P =* 0.02) in skeletal muscle of calves from the HME and LME groups compared to the CME group. In contrast, the mRNA expression of *MYH7* tended to be greater (*P* = 0.07) in the skeletal muscle of calves from LME compared to the CME group. An increased mRNA expression of both *MYH2a* (*P =* 0.04) and *MYH2 × *(*P *= 0.01) was observed in the skeletal muscle of calves from LME compared to the HME group (Table 6).

**Table 6.** Least-square means ± SEM for mRNA expression of metabolic energy markers in skeletal muscle of calves born to dams fed prepartum diets providing 118% (HME), 104% (CME), and 92% (LME) of the predicted metabolizable energy (ME) requirements from day −53 to calving, followed by an ad libitum postpartum lactation diet.

Item	Experimental treatments			*P*-value
	HME	CME	LME	Treatment
*Glucose metabolism*				
*IRS1*	5.50 ± 1.3	5.98 ± 1.43	4.87 ± 1.13	0.74
*SLC2A4*	6.28 ± 0.87	6.76 ± 1.01	6.47 ± 0.81	0.92
*Myosin heavy chain types*				
*MYH7*	4.18 ± 1.29	2.68 ± 0.83	5.25 ± 1.46	0.07
*MYH2a*	8.26 ± 1.53 ^b^	6.46 ± 1.21 ^ab^	4.51 ± 0.91 ^a^	0.04
*MYH2x*	2.59 ± 0.37 ^b^	3.48 ± 0.47 ^ab^	4.79 ± 0.59 ^a^	0.01
*MYH1*	3.81 ± 0.72 ^b^	6.60 ± 0.63 ^a^	3.82 ± 0.63 ^b^	0.02
*Oxidative metabolism*				
*PPARGC1α*	2.41 ± 0.87 ^b^	6.26 ± 0.87 ^a^	4.87 ± 0.87 ^a^	0.04
*PPARα*	0.84 ± 0.20 ^b^	1.65 ± 0.38 ^a^	1.43 ± 0.29 ^a^	0.04
*Fatty acid and lipid metabolism*				
*PPARγ*	2.49 ± 0.30	2.56 ± 0.30	2.53 ± 0.30	0.98
*ACACA*	0.85 ± 0.16	0.93 ± 0.16	1.16 ± 0.18	0.28
*CPT1α*	2.66 ± 0.94	3.22 ± 1.10	2.72 ± 0.85	0.80
*FASN*	1.63 ± 0.62	2.00 ± 0.77	1.66 ± 0.57	0.80
*SREBF1*	0.98 ± 0.39	1.02 ± 0.38	0.61 ± 0.22	0.19
*UCP3*	2.38 ± 0.65	2.1 ± 0.48	2.87 ± 0.58	0.45
*Muscle mass control*				
*MEF2A*	0.60 ± 0.14 ^b^	0.52 ± 0.12 ^b^	1.17 ± 0.24 ^a^	0.01

ACACA, Acetyl-CoA carboxylase; CPT1α, Carnitine palmitoyltransferase 1 alpha; FASN, fatty acid synthase; IRS1, Insulin receptor 1; MEF2A, myocyte enhancer factor 2A; MYH7, Myosin heavy-chain 7; MYH2a, Myosin heavy-chain 2a; MYH2x, Myosin heavy-chain 2x; MYH1, Myosin heavy-chain 1; PPARGC1α, peroxisome proliferator-activated receptor gamma coactivator 1-alpha; PPARα, peroxisome proliferator-activated receptor alpha; PPARγ, peroxisome proliferator-activated receptor gamma; SLC2A4, Glucose transporter 4; SREBF1, Sterol Regulatory Element-Binding Transcription Factor 1; UCP3, Uncoupling protein 3.

^b-b^Means within a row lacking a common superscript letter differ at *P* < 0.05.

A greater mRNA expression of *PPARα* (*P* = 0.04) and *PPARGC1α* (*P* = 0.04) was observed in the skeletal muscle of calves from the LME group compared to the HME group, without differences for *PPARγ* (*P* = 0.98; Table 6). For mRNA expression of markers of oxidative and lipid metabolism in skeletal muscle tissue of offspring, including *SREBF1 (P* = 0.19), *ACACA (P =* 0.28), *FASN (P =* 0.80), *CPT1α* (*P =* 0.80), and *UCP3* (*P =* 0.45), no differences were found among treatments (Table 6).

### AMPK and AKT activity

**Figure 1.**
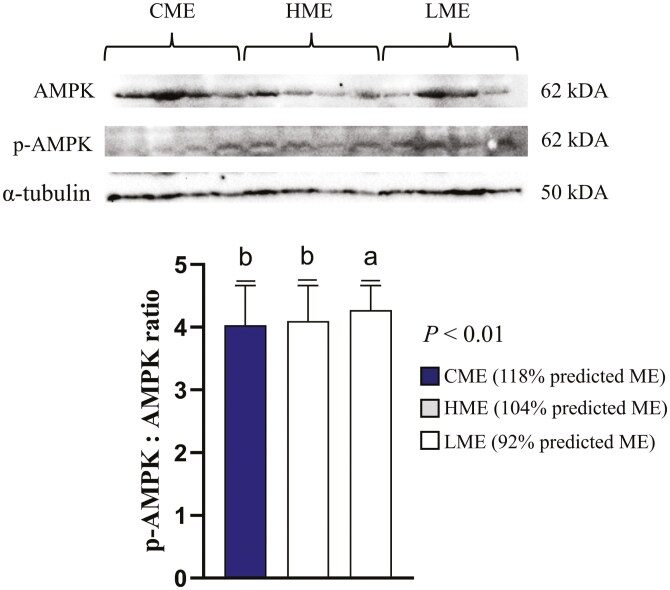
Least square means ± SEM of protein abundance of the ratio phospo-AMP-activated protein kinase (p-AMPK)/AMP-activated protein kinase (AMPK) in skeletal muscle of calves born to beef cows fed 104% (CME), 118% (HME), and 92% (LME) of predicted metabolizable energy intake (ME) at late gestation. Differences were considered when *P *≤ 0.05 and represented by different letters.

**Figure 2.**
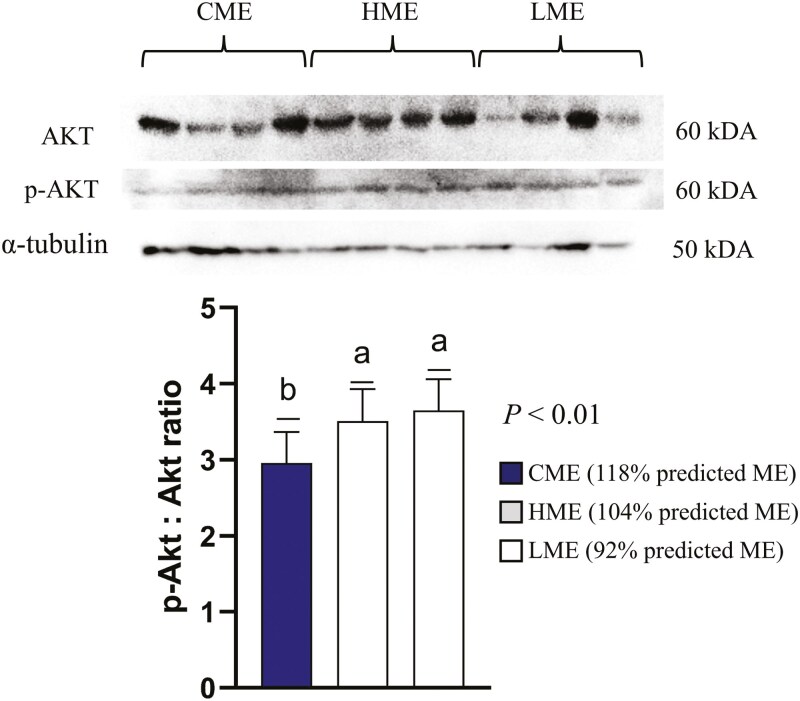
Least square means ± SEM of protein abundance of the ratio phospho-protein kinase B (p-Akt)/ protein kinase B (Akt) in skeletal muscle of calves born to beef cows fed 104% (CME), 118% (HME), and 92% (LME) of predicted metabolizable energy intake (ME) at late gestation. Differences were considered when *P *≤ 0.05 and represented by different letters.

The p-AMPK/AMPK abundance ratio was greater (*P *< 0.01) in the skeletal muscle of calves born to dams fed the LME diet compared to those born to dams fed CME and HME diets (Figure 1). In contrast, the abundance ratio of p-Akt/Akt was greater in the skeletal muscle of calves born to dams fed both the HME and LME diets compared to those born to dams fed the CME diet *(P* < 0.01; Figure 2).

## Discussion

The objective of the present study was to investigate how ME intake during late gestation influences the skeletal muscle energy metabolism of offspring in beef cattle. Our results demonstrated that variations in cow performance were impacted by the level of ME intake, validating the effectiveness of the experimental treatments. As intended, all cows were managed under a negative energy balance during late gestation. Although dietary treatments were formulated to provide 92%, 104%, and 118% of predicted ME requirements, it is important to note that the reference model used to estimate ME requirements assumed a 715 kg Angus cow with BCS 7, programmed to lose 2 BCS units over the last 100 d of gestation. Thus, even cows fed 118% of predicted requirements were not necessarily in positive energy balance. In fact, all cows, including those in the HME group, mobilized subcutaneous fat as objectively measured by ultrasound, confirming a negative or near-balanced energy state. Although HME and CME cows showed positive ADGs, these gains are largely attributable to gestational tissue accretion (e.g., fetus, uterus, fluids), which is estimated at approximately 0.5 kg/d in late gestation (Gionbelli et al., 2015). These differences in maternal performance were crucial for enabling the investigation of calf energy metabolism, which was the primary goal of this study. As such, the subsequent discussion will focus exclusively on the evidence supporting the notion that maternal ME intake may impact the energy metabolism of the offspring.

Previous studies have suggested that the effects of maternal nutrition can have long-lasting effects, during the calf’s early development until adulthood even when changes in birth weight are not observed (Lukaszewski et al., 2013; Du, 2023; Nascimento et al., 2024). In the current study, no differences in birth weight were observed among the treatment groups, indicating that overall fetal growth was not affected. However, the activity of AMPK (AMP-activated protein kinase), a key enzyme involved in regulating cellular energy metabolism, was influenced by maternal diet. AMPK is primarily activated under conditions of low ATP levels and an increase in AMP/ADP, playing a critical role in modulating muscle metabolism by promoting fatty acid oxidation and glucose uptake (Cantó and Auwerx, 2009) . In the current study, calves from the LME group showed higher AMPK activation compared to those from the HME and CME groups. This difference occurred despite all calves being managed as a single herd, under ad libitum feeding conditions, and with no significant variations in their blood metabolic profiles. This observation suggests that maternal ME intake, particularly at 92% of the required ME during late gestation, may affect the metabolic flexibility of skeletal muscle in the offspring.

AMPK regulates the expression of the transcription factors *PPARGC1α* and *PPARα*, which form a complex that promotes the expression of various genes involved in fatty acid oxidation and mitochondrial function (Liang and Ward, 2006). The greater mRNA expression of *PPARα* and *PPARGC1α* in the skeletal muscle of calves born to cows from the LME group compared to the HME group, along with greater AMPK activation, suggests the increase in beta-oxidation, despite the lack of differences in blood NEFA concentrations between these groups. Moreover, the *CPT1α* which is involved in promoting fatty acid oxidation for energy production, and *UCP3* which is involved in mitochondrial efficiency (Hoeks et al., 2006; Bougarne et al., 2018) are also both regulated by *PPARGC1α* and *PPARα* (Song et al., 2004; Villarroya et al., 2007). In our study, we did not identify differences for *CPT1α or UCP3* between the treatments. Therefore, our findings suggest that the higher expression of *PPARGC1α* and *PPARα* did not necessarily reflect an increase in the flux of long-chain fatty acids into mitochondria and protective effects due to this mechanism. Our findings partially corroborate those found by Aragão et al. (2014), where the offspring born to dams with protein restriction during pregnancy showed greater activation of AMPK and greater expression of *PPARGC1α*, *PPARα*, *CPT1α*, and *UCP3* in ad libitum conditions. In addition, the activation of AMPK and greater expression of *PPARGC1α* may lead to more efficient individuals in terms of animal productivity, since studies have indicated that high mitochondrial respiratory activity and AMPK phosphorylation are present in individuals with high feed efficiency (Bottje and Kong, 2013). Previous studies have shown that mitochondria from the muscles of more efficient animals (low residual feed intake) exhibit reduced production of reactive oxygen species compared to less efficient animals (with high residual feed intake; Grubbs et al., 2013). Additionally, the mitochondrial protein profile in more efficient animals revealed enhanced antioxidant defenses (Grubbs et al., 2013). This indicates a lower susceptibility to oxidative stress, reducing the need for cellular repair and enabling a greater allocation of dietary energy toward growth (Rauw et al., 2025). Furthermore, feed efficiency is closely linked to metabolic flexibility, the ability of an organism to adapt fuel oxidation to fuel availability, which allows for optimized nutrient utilization and energy allocation toward growth (Rauw et al., 2025). In this sense, our findings suggest that calves born to dams fed 92% of ME at late gestation have change the energy metabolism of their skeletal muscle by increasing the fatty acid oxidation pathway, which could lead to changes in animal performance later in life.

*PPARGC1α*, a key regulator of energy metabolism and mitochondrial biosynthesis in skeletal muscle, influences muscle development and fatty acid oxidation by modulating mitochondrial number and respiration (Hood et al., 2006). However, impaired mitochondrial function, reduced biogenesis, and increased oxidative damage can negatively affect muscle development (Zou et al., 2016). Notably, calves born to cows that consumed 118% of their ME requirements during late gestation exhibited reduced *PPARGC1α* expression. This observation aligns with findings from Zou et al. (2017), which reported that the expression of several mitochondrial function-related genes was significantly lower in fetuses from overfed dams, suggesting compromised skeletal muscle mitochondrial function. Furthermore, mitochondrial dysfunction resulting from maternal overnutrition has been associated with increased oxidative stress in offspring. This increased oxidative stress can further impair growth by diverting energy away from muscle development and toward cellular repair mechanisms, thereby reducing the availability of dietary energy for productive purposes (Zou et al., 2017). In addition, the reduced expression of *PPARGC1α* leads to lower muscle mass and a higher proportion of small myofibers (Ma et al., 2022). Given its essential role in muscle metabolism, variations in *PPARGC1α* expression can therefore directly impact growth efficiency.

The *PPARGC1α* is linked to muscle tissue plasticity, with higher mRNA expression associated with the transition from glycolytic to oxidative muscle fibers (Lin et al., 2002). In the LME group, which showed elevated mRNA expression of *PPARGC1α* in skeletal muscle, a higher proportion of oxidative muscle fibers was expected compared to the HME group. However, no differences in mRNA expression of *MYH7* were observed between the groups, though both LME and HME groups showed greater mRNA expression of *MYH7* compared to the CME group. Additionally, the lower mRNA expression of *MYH1* in both the LME and HME groups compared to the CME group suggests that meeting 92% and 118% of maternal predicted ME requirements can trigger mechanisms that alter skeletal muscle fuel usage. A greater mRNA expression of *MYH2a* and *MYH2x* was found in the LME group compared to the HME group. These genes are responsible for type IIa muscle fibers, which can switch between glycolytic and oxidative fuel use (Norman et al., 2012). Maternal undernutrition has been shown to decrease type II muscle fibers and increase type muscle I fibers in ewes (Fahey et al., 2005) . Our findings suggest a similar mechanism in calves born to dams from the high ME group, with a reduction in type IIa muscle fibers and impaired oxidative metabolism, indicating reduced metabolic flexibility in the skeletal muscle of calves from this group.

Another key regulator of muscle energy metabolism is the protein kinase B (Akt) pathway, which plays a critical role in promoting protein synthesis (Schiaffino et al., 2013) . One of the ways Akt is activated is through insulin and its receptor, which can stimulate glucose uptake via SLC2A4 (Long et al., 2006) . In our study, the insulin and glucose blood levels, and the mRNA expression of insulin receptor subunit 1 (*IRS1*) and *SLC2A4* did not differ among the groups, suggesting another pathway might be responsible for Akt activation in the HME group. Although AMPK and Akt were initially thought to have an antagonistic relationship, recent studies suggest that chronic AMPK activation can trigger Akt activation as a compensatory mechanism to regulate protein synthesis (Shoemaker et al., 2023). This may explain the increased Akt activation observed in the LME group in our study. In fact, in association with increased Akt activation, we also observed an increased mRNA expression for *MEF2A* in the LME group compared to CME and HME. The *MEF2A* (myocyte enhancer factor isoform 2A) is an important transcription factor involved in muscle mass control and hypertrophy (Schiaffino et al., 2021), and can be regulated by Akt and AMPK to further control muscle development (Chen et al., 2020). Overall, the increased Akt activation and mRNA expression of MEF2A in the skeletal muscle of calves born to dams in the LME group may indicate a compensatory mechanism in response to elevated AMPK activation, helping to mitigate the effects of reduced anabolic processes caused by AMPK activation.

## Conclusion

Our findings suggest that calves born to dams fed 92% of the predicted ME requirement show a metabolic adaptation that favors fatty acid oxidation for muscle energy production. The greater mRNA expression of markers for type I and IIa muscle fibers in agreement with increased expression of oxidative metabolism genes may provide an advantage in terms of energy flexibility and future productive efficiency. Conversely, calves born to dams fed 118% of the predicted ME requirement decreased expression of oxidative metabolism markers, suggesting potential impairments in mitochondrial function and metabolic flexibility in skeletal muscle. Lastly, our findings suggest that excessive maternal energy intake can cause mitochondrial dysfunction and elevate oxidative stress in the offspring’s skeletal muscle, which may ultimately hinder muscle development and growth efficiency later in life.

## Abbreviations:

ACACA: acetyl-CoA carboxylase alpha
ADF: acid detergent fiber
ADG: average daily gain;
Akt: , protein kinase B
AMPK: AMP-activated protein kinase
BCS: body condition score
BHBA: beta-hydroxybutyrate
BW: body weight
CP: crude protein
*CPT1α*: , carnitine palmitoyltransferase 1A
DM: dry matter
EE: crude fat
FASN: fatty acid synthase
FBW: final body weight
FSFT: final subcutaneous fat thickness
IBW: initial body weight
IRS1: , insulin receptor substrate 1
ISFT: initial subcutaneous fat thickness
ME: metabolizable energy
MEF2A: myocyte enhancer factor isoform 2A
MYH1: , myosin heavy chain 1
MYH2a: , myosin heavy chain 2a
MYH2x: myosin heavy chain 2x
MYH7: myosin heavy chain 7
NDF: Neutral detergent fiber
NEFA: Non-esterified-fatty-acids
OBRC: Ontario Beef Research Center
PPARα: peroxisome proliferator activated receptor alpha
PPARγ: peroxisome proliferator activated receptor gamma
PPARGC1α: peroxisome proliferator active receptor gamma coactivator 1 alpha
p-Akt: , phospho-protein kinase B
p-AMPK: phospo-AMP-activated protein kinase
SREBF1: sterol regulatory element binding transcription factor 1
SLC2A4: , solute carrier family 2 member 4
UCP3: uncoupling protein 3
VBW: variation of body weight
VSFT: variation subcutaneous fat thickness

## References

[CIT0040] AOAC. 1990. Official methods of analysis. 15th ed. Arlington (VA): Association of Official Analytical Chemists.

[CIT0001] Aragão, R. S., O. Guzmán-Quevedo, G. Pérez-García, R. Manhães-De-Castro, and F. Bolaños-Jiménez. 2014. Maternal protein restriction impairs the transcriptional metabolic flexibility of skeletal muscle in adult rat offspring. Br. J. Nutr. 112:328–337. doi: https://doi.org/10.1017/S0007114514000865 24823946

[CIT0002] Barcelos, S. de S, K. B. Nascimento, T. E. da Silva, R. Mezzomo, K. S. Alves, M. de Souza Duarte, and M. P. Gionbelli. 2022. The effects of prenatal diet on calf performance and perspectives for fetal programming studies: a meta-analytical investigation. Animals 12:2145. doi: https://doi.org/10.3390/ani12162145 36009734 PMC9404886

[CIT0003] Bottje, W., and B. W. Kong. 2013. Cell biology symposium: feed efficiency: mitochondrial function to global gene expression. J. Anim. Sci. 91:1582–1593. doi: https://doi.org/10.2527/jas.2012-5787 23148240

[CIT0004] Bougarne, N., B. Weyers, S. J. Desmet, J. Deckers, D. W. Ray, B. Staels, and K. De Bosscher. 2018. Molecular actions of PPARα in lipid metabolism and inflammation. Endocr Rev. 39:760–802. doi: https://doi.org/10.1210/er.2018-00064 30020428

[CIT0041] Cantó, C., and J. Auwerx. 2009. PGC-1α, SIRT1 and AMPK, an energy sensing network that controls energy expenditure Curr. Opin. Lipidol. 20(2):98–105. doi: https://doi.org/10.1097/MOL.0b013e328328d0a4 19276888 PMC3627054

[CIT0005] Carvalho, E. B., T. C. Costa, L. P. Sanglard, K. B. Nascimento, J. A. M. Meneses, M. C. Galvão, N. V. L. Serão, M. S. Duarte, and M. P. Gionbelli. 2022. Transcriptome profile in the skeletal muscle of cattle progeny as a function of maternal protein supplementation during mid-gestation. Livest. Sci. 263:104995. doi: https://doi.org/10.1016/j.livsci.2022.104995

[CIT0038] CCAC. 1993. Guide to the use of experimental animals. Vol. 1. Ottawa (ON): Canadian Council on Animal Care.

[CIT0007] Chen, M., C. Ji, Q. Yang, S. Gao, Y. Peng, Z. Li, X. Gao, Y. Li, N. Jiang, Y. Zhang, et al. 2020. AKT2 regulates development and metabolic homeostasis via AMPK-depedent pathway in skeletal muscle. Clin. Sci, 134:2381–2398. doi: https://doi.org/10.1042/CS20191320 32880392

[CIT0006] Chen, H., C. Wang, S. Huasai, and A. Chen. 2022. Effect of prepartum dietary energy density on beef cow energy metabolites, and birth weight and antioxidative capabilities of neonatal calves. Sci. Rep. 12:4828. doi: https://doi.org/10.1038/s41598-022-08809-6 35318381 PMC8941139

[CIT0009] Costa, T. C., T. A. O. Mendes, M. M. S. Fontes, M. M. Lopes, M. Du, N. V. L. Serão, L. M. P. Sanglard, F. Bertolini, M. F. Rothschild, F. F. Silva, et al. 2021b. Transcriptome changes in newborn goats’ skeletal muscle as a result of maternal feed restriction at different stages of gestation. Livest. Sci. 248:104503. doi: https://doi.org/10.1016/j.livsci.2021.104503

[CIT0010] Du, M. 2023. Prenatal development of muscle and adipose and connective tissues and its impact on meat quality. Meat Muscle Biol. 7:16230. doi: https://doi.org/10.22175/mmb.16230

[CIT0042] Fahey, A. J., J. M. Brameld, T. Parr, and P. J. Buttery. 2005. The effect of maternal undernutrition before muscle differentiation on the muscle fiber development of the newborn lamb1,2 J. Anim. Sci. 83(11):2564–2571. doi: https://doi.org/10.2527/2005.83112564x 16230653

[CIT0011] Funston, R. N., D. M. Larson, and K. A. Vonnahme. 2010. Effects of maternal nutrition on conceptus growth and offspring performance: implications for beef cattle production. J. Anim. Sci. 88:E205–E215. doi: https://doi.org/10.2527/jas.2009-2351 19820049

[CIT0012] Gionbelli, M. P., M. S. Duarte, S. C. Valadares Filho, E. Detmann, M. L. Chizzotti, F. C. Rodrigues, T. R. S. Gionbelli, M. I. Marcondes, and M. G. Machado. 2015. Achieving body weight adjustments for feeding status and pregnant or non-pregnant condition in beef cows. PLoS One 10:e0112111. doi: https://doi.org/10.1371/journal.pone.0112111 25793770 PMC4368534

[CIT0013] Grubbs, J. K., A. N. Fritchen, E. Huff-Lonergan, N. K. Gabler, and S. M. Lonergan. 2013. Selection for residual feed intake alters the mitochondria protein profile in pigs. J. Proteomics 80:334–345. doi: https://doi.org/10.1016/j.jprot.2013.01.017 23403255

[CIT0039] Higgs, R. J., L. E. Chase, D. A. Ross, and M. E. Van Amburgh. 2015. Updating the Cornell Net Carbohydrate and Protein System feed library and analyzing model sensitivity to feed inputs J. Dairy Sci. 98(9):6340–6360. doi: https://doi.org/10.3168/jds.2015-9379 26142848

[CIT0014] Hoeks, J., M. K. C. Hesselink, A. P. Russell, M. Mensink, W. H. M. Saris, R. P. Mensink, and P. Schrauwen. 2006. Peroxisome proliferator-activated receptor-γ coactivator-1 and insulin resistance: acute effect of fatty acids. Diabetologia 49:2419–2426. doi: https://doi.org/10.1007/s00125-006-0369-2 16896940

[CIT0015] Hood, D. A., I. Irrcher, V. Ljubicic, and A. -M. Joseph. 2006. Coordination of metabolic plasticity in skeletal muscle. J. Exp. Biol. 209:2265–2275. doi: https://doi.org/10.1242/jeb.02182 16731803

[CIT0016] Liang, H., and W. F. Ward. 2006. Staying Current PGC-1: a key regulator of energy metabolism. Adv. Physiol. Educ 30:145–151. doi: https://doi.org/10.1152/advan.00052.2006 17108241

[CIT0017] Lin, J., H. Wu, P. T. Tarr, C. -Y. Zhang, Z. Wu, O. Boss, L. F. Michael, P. Puigserver, E. Isotani, E. N. Olson, et al. 2002. Transcriptional co-activator PGC-1α drives the formation of slow-twitch muscle fibres. Nature 418:797–801. doi: https://doi.org/10.1038/nature00904 12181572

[CIT0044] Long, Y. C., and J. R. Zierath. 2006. AMP-activated protein kinase signaling in metabolic regulation. J. Clin. Invest. 116:1776–1783. doi:10.1172/JCI29044 16823475 PMC1483147

[CIT0018] Lukaszewski, M. -A., D. Eberlé, D. Vieau, and C. Breton. 2013. Nutritional manipulations in the perinatal period program adipose tissue in offspring. Am. J. Physiol. Endocrinol. Metab. 305:E1195–E1207. doi: https://doi.org/10.1152/ajpendo.00231.2013 24045869

[CIT0019] Ma, M., B. Cai, S. Kong, Z. Zhou, J. Zhang, X. Zhang, and Q. Nie. 2022. PPARGC1A is a moderator of skeletal muscle development regulated by miR-193b-3p. Int. J. Mol. Sci. 23:9575. doi: https://doi.org/10.3390/ijms23179575 36076970 PMC9455960

[CIT0021] Meneses, J. A. M., K. B. Nascimento, M. C. Galvão, G. M. Moreira, L. H. L. Chalfun, S. P. de Souza, G. D. Ramírez-Zamudio, M. M. Ladeira, M. S. Duarte, D. R. Casagrande, et al. 2024. Protein supplementation during mid-gestation affects maternal voluntary feed intake, performance, digestibility, and uterine blood flow of beef cows. J. Anim. Physiol. Anim. Nutr. (Berl) 108:1678. doi: https://doi.org/10.1111/jpn.14001 38922982

[CIT0022] Nascimento, K. B., M. C. Galvão, J. A. M. Meneses, G. D. Ramírez-Zamudio, D. G. Pereira, P. V. R. Paulino, D. R. Casagrande, T. R. S. Gionbelli, M. M. Ladeira, M. S. Duarte, et al. 2024. Maternal protein supplementation during mid-gestation improves offspring performance and metabolism in beef cows. J. Anim. Sci. 102:skae058. doi: https://doi.org/10.1093/jas/skae058 38437631 PMC10998463

[CIT0023] Norman, A. M., J. L. Miles-Chan, N. M. Thompson, B. H. Breier, and K. Huber. 2012. Postnatal development of metabolic flexibility and enhanced oxidative capacity after prenatal undernutrition. Reproduct. Sci. 19:607–614. doi: https://doi.org/10.1177/1933719111428519 22138545

[CIT0024] Rauw, W. M., L. H. Baumgard, and J. C. M. Dekkers. 2025. Review: feed efficiency and metabolic flexibility in livestock. Animal. 19:101376. doi: https://doi.org/10.1016/j.animal.2024.101376 39673819

[CIT0025] Sanglard, L. P., M. Nascimento, P. Moriel, J. Sommer, M. Ashwell, M. H. Poore, M. D. S. Duarte, and N. V. L. Serão. 2018. Impact of energy restriction during late gestation on the muscle and blood transcriptome of beef calves after preconditioning. BMC Genomics 19:702. doi: https://doi.org/10.1186/s12864-018-5089-8 30253751 PMC6156876

[CIT0026] Santos, M. M., T. C. Costa, G. D. Ramírez-Zamudio, K. B. Nascimento, M. P. Gionbelli, and M. de Souza Duarte. 2022. Prenatal origins of productivity and quality of beef. Rev. Bras. Zootec. 51:e20220061. doi: https://doi.org/10.37496/RBZ5120220061

[CIT0043] Schiaffino, S., K. A. Dyar, S. Ciciliot, B. Blaauw, and M. Sandri. 2013. Mechanisms regulating skeletal muscle growth and atrophy FEBS J. 280(17):4294–4314. doi: https://doi.org/10.1111/febs.12253 23517348

[CIT0027] Schiaffino, S., C. Reggiani, T. Akimoto, and B. Blaauw. 2021. Molecular mechanisms of skeletal muscle hypertrophy. J. Neuromuscul. Dis. 8:169–183. doi: https://doi.org/10.3233/JND-200568 33216041 PMC8075408

[CIT0028] Shoemaker, M. E., Z. M. Gillen, D. H. Fukuda, and J. T. Cramer. 2023. Metabolic flexibility and inflexibility: pathology underlying metabolism dysfunction. J. Clin. Med. 12:4453. doi: https://doi.org/10.3390/jcm12134453 37445488 PMC10342527

[CIT0029] Song, S., Y. Zhang, K. Ma, L. Jackson-Hayes, E. N. Lavrentyev, G. A. Cook, M. B. Elam, and E. A. Park. 2004. Peroxisomal proliferator activated receptor gamma coactivator (PGC-1α) stimulates carnitine palmitoyltransferase I (CPT-Iα) through the first intron. Biochim. Biophys. Acta. 1679:164–173. doi: https://doi.org/10.1016/j.bbaexp.2004.06.006 15297149

[CIT0030] Steibel, J. P., R. Poletto, P. M. Coussens, and G. J. M. Rosa. 2009. A powerful and flexible linear mixed model framework for the analysis of relative quantification RT-PCR data. Genomics 94:146–152. doi: https://doi.org/10.1016/j.ygeno.2009.04.008 19422910

[CIT0031] Talbot, J., and L. Maves. 2016. Skeletal muscle fiber type: using insights from muscle developmental biology to dissect targets for susceptibility and resistance to muscle disease. Wiley. Interdiscip. Rev. Dev. Biol. 5:518–534. doi: https://doi.org/10.1002/wdev.230 27199166 PMC5180455

[CIT0032] Van Amburgh, M. E., A. Foskolos, and R. J. Higgs. 2015a. Balancing diets with the CNCPS v6.5 – What’s changed and implications for use. Proc. Cornell Nutrition Conference, Syracuse, NY, Cornell University (2015), pp. 115–122.

[CIT0033] Van Amburgh, M. E., E. A. Collao-Saenz, R. J. Higgs, D. A. Ross, E. B. Recktenwald, E. Raffrenato, L. E. Chase, T. R. Overton, J. K. Mills, and A. Foskolos. 2015b. The cornell net carbohydrate and protein system: updates to the model and evaluation of version 6.5. J. Dairy Sci. 98:6361–6380. doi: https://doi.org/10.3168/jds.2015-9378 26142847

[CIT0034] Villarroya, F., R. Iglesias, and M. Giralt. 2007. PPARs in the control of uncoupling proteins gene expression. PPAR Res. 2007:1–12. doi: https://doi.org/10.1155/2007/74364 PMC1779581 17389766

[CIT0035] Waldon, N., K. Nickles, A. Parker, K. Swanson, and A. Relling. 2023. A review of the effect of nutrient and energy restriction during late gestation on beef cattle offspring growth and development. J. Anim. Sci. 101:skac319. doi: https://doi.org/10.1093/jas/skac319 36592744 PMC9831102

[CIT0037] Zou, T., D. He, B. Yu, J. Yu, X. Mao, P. Zheng, J. He, Z. Huang, Y. Shu, Y. Liu, et al. 2016. Moderately increased maternal dietary energy intake delays foetal skeletal muscle differentiation and maturity in pigs. Eur. J. Nutr. 55:1777–1787. doi: https://doi.org/10.1007/s00394-015-0996-9 26179476

[CIT0036] Zou, T. D., B. Yu, J. Yu, X. B. Mao, P. Zheng, J. He, Z. Q. Huang, D. T. He, and D. W. Chen. 2017. Mitochondrial biogenesis is decreased in skeletal muscle of pig fetuses exposed to maternal high-energy diets. Animal 11:54–60. doi: https://doi.org/10.1017/S1751731116001269 27349347

